# Periostin in tumor microenvironment is associated with poor prognosis and platinum resistance in epithelial ovarian carcinoma

**DOI:** 10.18632/oncotarget.6700

**Published:** 2015-12-21

**Authors:** Pi-Lin Sung, Yi-Hua Jan, Shih-Chieh Lin, Chao-Cheng Huang, Hao Lin, Kuo-Chang Wen, Kuan-Chong Chao, Chiung-Ru Lai, Peng-Hui Wang, Chi-Mu Chuang, Hua-Hsi Wu, Nae-Fang Twu, Ming-Shyen Yen, Michael Hsiao, Chi-Ying F. Huang

**Affiliations:** ^1^ Institute of Clinical Medicine, National Yang-Ming University, Taipei, Taiwan; ^2^ Department of Obstetrics and Gynecology, Taipei Veterans General Hospital, Taipei, Taiwan and School of Medicine, National Yang-Ming University, Taipei, Taiwan; ^3^ Genomics Research Center, Academia Sinica, Taipei, Taiwan; ^4^ Department of Pathology, Taipei Veterans General Hospital, Taipei, Taiwan; ^5^ Department of Pathology, Kaohsiung Chang Gung Memorial Hospital and Chang Gung University College of Medicine, Kaohsiung, Taiwan; ^6^ Department of Obstetrics and Gynecology, Kaohsiung Chang Gung Memorial Hospital and Chang Gung University College of Medicine, Kaohsiung, Taiwan; ^7^ Institute of Biopharmaceutical Sciences, National Yang-Ming University, Taipei, Taiwan

**Keywords:** periostin, platinum resistance, biomarker, microenvironment, epithelial ovarian cancer

## Abstract

The interplay between tumor microenvironment and cancer that causes chemoresistance remains unclear. By analyzing public available microarray datasets, we identified that periostin (POSTN) was overexpressed in cancer stroma in epithelial ovarian cancer (EOC) patients. Immunohistochemistry analysis showed overexpression of stromal POSTN is a powerful independent poor prognostic predictor for EOC patients. Furthermore, patients with high levels of stromal POSTN tend to have higher percentage of cisplatin resistance compared to those with low levels of stromal POSTN. Moreover, we found POSTN treatment can induce cisplatin resistant and activate AKT pathway in A2780 cells *in vitro*. Inhibition of AKT activity by AKT inhibitor MK-2206 abolished POSTN-induced AKT activation and cisplatin resistance *in vitro*. Taken together, we found high POSTN expression in cancer microenvironment is correlated with poor prognosis in EOC patients and associated with platinum resistance. The effect of POSTN in cancer stroma cells may activate AKT pathway in tumor and AKT inhibitor can be beneficial to augment the efficacy of existing cancer therapeutics.

## INTRODUCTION

Epithelial ovarian cancer (EOC) is the third most common type of gynecologic cancer in the United States but a leading cause of gynecologic cancer deaths. It is estimated that 21,980 new cases were diagnosed in 2014, with 14,270 deaths [1]. Although aggressive surgical cytoreduction followed by standard platinum-taxene chemotherapy generally improved the survival rate of EOC patients, the 5-year survival rate remains dismal (∼30%) [2]. Platinum-resistance, defined as relapse within 6 months, is one of the major causes for the low survival rate. It is also a big challenge for successful treatment and a reference for deciding second-line treatments [3]. Therefore, predictive biomarkers for platinum-resistance in EOC patients are desperately needed.

Periostin (POSTN, formerly called 改成 also known as osteoblast-specific factor-2) is a disulfide-liked 90-kDa secretary protein expressed in the periosteum in bone tissues as well as an extracellular matrix (ECM) protein [4–6]. POSTN is up-regulated in a wide variety of cancer, such as colon, pancreatic, breast, head and neck, thyroid, and gastric cancer, non-small cell lung cancer and neuroblastoma [4, 7–12]. POSTN mRNA levels is also upregulated in ovarian tumors [13], and its expression in ovarian tumor cells was correlated with clinical late stage and tumor recurrence [14]. EOC cells can secret POSTN and accumulate in malignant ascites of ovarian cancer patients [11]. Moreover, recombinant purified POSTN supports the adhesion and migration of ovarian epithelial cancer cells by interacting with integrin receptors αVβ3 and αVβ5 [11]. POSTN also increases tumor angiogenesis and decreases tumor cell apoptosis in EOC [14]. Neutralizing monoclonal antibody against POSTN was shown to inhibit anchorage-independent growth and survival of POSTN-expressing cells and its neutralizing effects also suppress cancer cell migration and invasion [15]. Expression of POSTN in ovarian tumor cells was correlated significantly with advanced stage and disease recurrence [14]. However, an increasing number of evidences showed that POSTN expressed in the cancer microenvironment was also associated with poor prognosis in various types of cancer such as non-small cell lung cancer [16], pancreatic cancer [17, 18], prostate cancer [19] as well as ovarian cancer [20, 21]. Therefore, the histological localization of POSTN expression that really impacts patient survival remains controversial.

POSTN can activate the PI3K/AKT signaling pathway through α_v_β_3_ integrin to increase cellular survival in colon cancer [22] and through α_6_β_4_ integrin complex to promote invasiveness and resistance of pancreatic cancer cells to hypoxia-induced cell death [17]. In EOC, recombinant POSTN can also stimulate Akt phosphorylation to increase resistance to paclitaxel [23]. Molecular actions in AKT pathway were shown to be an important determinant of chemosensitivity to platinum in EOC [24–26]. However, the relationship between POSTN expression and platinum resistance has not been studied intensively in EOC.

In this study, we used tissue microarray that consists 308 epithelial ovarian cancer patients to analyze the prognostic value of POSTN and its association with chemo-response and clinical outcome.

## RESULTS

### Identification of up-regulated POSTN in ovarian cancer stroma

**Figure 1:**
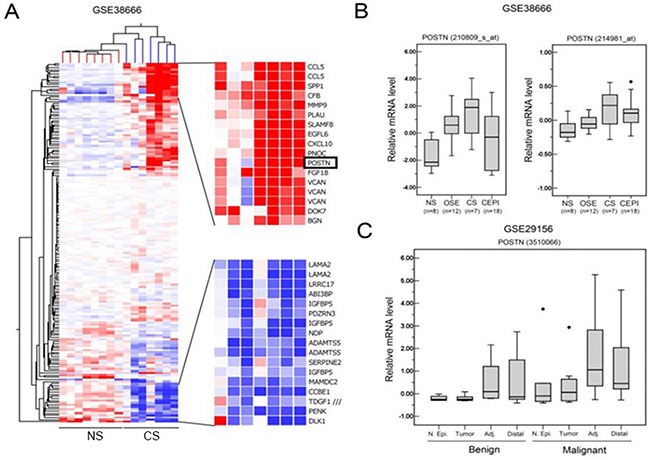
POSTN is upregulated in stroma of epithelial ovarian adenocarcinoma **A.** Clustering analysis of differentially expressed genes that are annotated to be expressed in extracellular space between normal stroma (NS) and cancer stroma (CS). **B.** Relative *POSTN* levels in normal stroma (NS), normal ovarian surface epithelium (OSE), cancer stroma (CS), and cancer epithelium (CEPI) in GSE38666. **C.** Relative *POSTN* levels in normal epithelium (N. Epi), tumor, adjacent cancer stroma (Adj), and distal cancer stroma (distal) between benign tumor and malignant tumor in GSE29156.

To determine gene expression signatures in different histological localization of ovarian cancer, we analyzed microarray datasets from GSE38666 that has gene expression profiles of 7 cancer stroma (CS) samples and 18 cancer epithelium samples (CEPI), along with 8 normal stroma (NS) samples and 12 normal ovarian surface epithelium (OSE) samples. Unsupervised hierarchical clustering of the microarray data found that the POSTN was overexpressed in CS along with other cytokines, growth factors and ECM components compared to NS (Figure 1A). Furthermore, POSTN mRNA expression was compared among NS (*n* = 8), OSE (*n* = 12), CS (*n* = 7), and CPEI (*n* = 18). The result showed that CS expressed the highest POSTN mRNA across all groups (Figure 1B). In another microarray datasets (GSE29156) that contain laser capture micro-dissected normal epithelium samples, tumor samples, adjacent tumor stroma samples and distal tumor stroma samples from both benign and malignant ovarian tumor, we found POSTN mRNA expression of malignant adjacent cancer stroma (Adj) was also the highest among all (Figure 1C).

### Stronger association of POSTN expression in cancer stroma and poor prognosis compared to tumor cells

**Figure 2:**
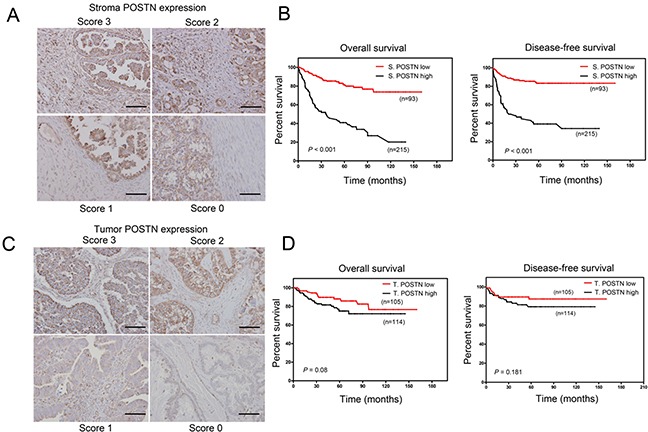
Overexpression of stromal POSTN predicts worse survival compared to tumor POSTN in EOC and borerline cancer patients **A.** Representative IHC staining of stroma POSTN expression scores. Scale bar: 100 μm. **B.** Kaplan–Meier analysis of overall survival (OS) and diseases-free survival (DFS) in 308 EOC patients stratified by high periostin expression (score 3) and low periostin expression (score 0-2) in tumor stroma. **C.** Representative IHC staining of tumor POSTN expression scores. Scale bar: 100 μm. **D.** Kaplan–Meier analysis of overall survival (OS) and diseases-free survival (DFS) in 308 EOC patients stratified by high periostin expression (score 3) and low periostin expression (score 0-2) in tumor.

To determine the prognostic value of POSTN form different histological location, we performed immunohistochemistry (IHC) analysis using tissue array that consists 308 EOC patients. Protein expression of POSTN can be observed in the cancer stroma and cancer cells. The staining expression was semiquantatively and arbitrarily score ranged from 0 (negative expression), 1 (weak expression), 2 (moderate expression) to 3 (strong expression) modified from Choi et al [20] (designed as 0, no staining or less than 10% of stromal or tumor cells ; 1, weak, 10-30% of stromal or tumor cells ; 2, 30-60% of stromal or tumor cells; 3, more than 60% of stromal or tumor cells). Then we divided patients into high POSTN expression as score 3 and low POSTN expression as score 0-2 (Figure 2A and 2C).

Table 1 summarized the number of patients with high POSTN expression in cancer stromal cells was 93 (30.2 %) and that with low POSTN expression was 215 (69.8%). The mean age of the patients was 53.96 ± 13.08 years old (range: 13-85 years old). The mean duration of follow-up was 48.5 ± 35.1 months (range: 1.0-160 months). The patients with strong POSTN staining in tumor-associated stroma had higher ratio of advanced FIGO stage compared with patients with low POSTN expression in cancer stroma (41.4% vs 20.6% ; *p* < 0.001). More optimal surgery were achieved in patients with low POSTN expression in cancer stromal cells (*p* = 0.025). There was no difference in age (*p* = 0.202), histological subgroups (*p* = 0.813), tumor grading (*p* = 0.747) and level of ca-125 pre-operatively (*p* = 0.359) or after 1st chemotherapy (*p* = 0.175) between the two groups. High stroma POSTN expression was associated with higher prevalence of high POSTN expression tumor cells (correlation coefficient = 0.219; *p* < 0.001). Also, patients with high POSTN expression in cancer stroma had higher tumor recurrent rate than patients with low POSTN expression in cancer stroma after first treatment (52.7% and 25.6%, *p* < 0.001).

**Table 1:** Pathological characteristics of patients with low and high stromal periostin expression

Characteristics	Patients with low stromal periostin expression (*n* = 215, 69.8%)	Patients with high stromal periostin expression (*n* = 93, 30.2 %)	*p*-value
**Age(y/o)**	53.4 ± 13.6	55.3 ± 11.7	0.202
FIGO stage (*n* = 300)* (%)			
Early stage (I-II)	127 (79.4)	33 (20.6)	<0.001^§^
Late stage (III-IV)	82(56.8)	58 (41.4)	
**Histopathologic subtype(%)**			
Borderline	12(85.7)	2(14.3)	0.813
Serous	67 (67.7)	32 (32.3)	
Mucinous	34 (70.8)	14 (29.2)	
Clear cell	45 (71.4)	18 (28.6)	
Endometrioid	42 (68.9.0)	19 (31.1)	
Others	15(65.2)	8(34.8)	
Grade (*n* = 184)*(%)			
Well to moderate (I-II)	50(66.7)	25(33.3)	0.747
Poor (III)	76(69.7)	33(30.3)	
Surgery (*n* = 156)*(%)			
Optimal	88(73.7)	32(26.7)	0.025^§^
Suboptimal	19(52.8)	17(47.2)	
**Ca-125 value**			
Pre-operative (*n* = 210)*	961 ± 3606	1352 ± 2457	0.359
Post-1st chemotherapy (*n* = 278)*	107 ± 251	223 ± 758	0.175
**Tumor periostin expression**			
Low score (0-2)	101 (82.1)	22 (17.9)	<0.001^$^
High score (3)	114(61.6)	71(38.4)	
**Tumor recurrence (%)**			
Yes	55 (25.6)	49(52.7)	<0.001^§^
No	160 (74.4)	44(47.3)	

* Patients numbers used to analyze in this study

§ Two-sided Fisher's exact test

$ Pearson correlation coefficient = 0.219; *p* < 0.001

**Figure 3:**
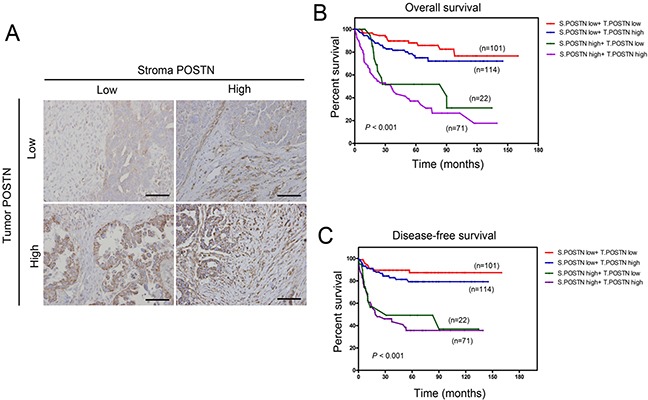
Overall and diseases-free survival analysis stratified by combination of stromal and tumor POSTN status **A.** Representative IHC staining of POSTN status in tumor and stroma. Scale bar: 100 μm. **B.** Kaplan Meier overall survival analysis stratified by combination of stromal and tumor POSTN status. **C.** Kaplan Meier disease-free survival analysis stratified by combination of stromal and tumor POSTN status.

In the analysis of clinical outcome, patients with a high POSTN expression in cancer stroma (n=93) had a mean disease-specific overall survival (OS) of 58.2 ± 6.2 months [95% confidence interval (95% CI), 46.3-70.4 months] and disease-specific progression-free survival (PFS) of 58.3 ± 7.2 months [95% CI, (44.4-72.6) months], whereas those with low POSTN expression in cancer stroma (*n* = 215) had a mean disease-specific OS of 129.1 ± 4.7 months (95% CI, 119.9-138.3 months) and mean PFS of 117 ± 5.0 months (95% CI, 107.3-126.8). Patients with high stromal POSTN expression had significantly lower OS and PFS than those with low stromal POSTN expression (both *p* < 0.001) (Figure 2B). On the other hands, patients with high POSTN expression in tumor (*n* = 114) did not show significant prognostic value in OS and PFS compared to patients with low POSTN expression in tumor (Figure 2D). Next, we performed survival analysis according to various combinations of tumor POSTN and stromal POSTN status (Figure 3A). The results showed patients with high POSTN expression in both stroma and tumor had the shortest OS and PFS among other groups (Figure 3B and 3C). Furthermore, multivariate Cox regression analysis showed that stromal POSTN levels and platinum resistance were found to be the only two significant predictors of patient outcome as evaluated by FPS (Table 2) or OS (Table 3).

**Table 2:** Univariants and multivariate analysis of disease-specific progression-free survival

Parameters	Comparison	Univariate analysis		Multivariate analysis	
		HR (95% CI)	*P*-value	HR (95% CI)	*P*-value
**Tumor periostin**	Low (0-2); High (3)	1.66(1.09-2.52)	0.018	0.80(0.37-1.76)	0.586
**Stromal periostine**	Low (0-2); High (3)	2.99(2.02-4.42)	<0.001	2.64(1.08-6.43)	0.033^§^
**Histologic grade**	G1-2; G3	1.90(1.10-3.30)	0.021	1.79(0.77-3.65)	0.195
**FIGO stage**	pT1-pT2; pT3-pT4	4.92(3.20-7.56)	<0.001	1.675(0.84-3.82)	0.135
**Platinum resistance**	No ; Yes	16.9(8.85-32.1)	<0.001	6.874(2.82-16.7)	<0.001^§^

Note: Cox proportional hazards regression was used to perform uni- and multivariant analysis for potentially important variants.

Abbreviations: HR, hazard ratio; CI, confidence interval.

§ Two-sided Cox proportional hazards regression using normal approximation.

**Table:** Univariants and multivariate analysis of disease-specific overall survival

Parameters	Comparison	Univariate analysis		Multivariate analysis	
		HR (95% CI)	*P*-value	HR (95% CI)	*P*-value
**Tumor periostin**	Low (0-2); High (3)	2.219(1.39-3.54)	0.001	1.09(0.47-2.57)	0.834
**Stromal periostine**	Low (0-2); High (3)	4.729(3.11-7.18)	<0.001	3.02(1.27-7.19)	0.012^§^
**Histologic grade**	G1-2; G3	1.824(1.06-3.31)	0.048	1.64(0.69-3.93)	0.263
**FIGO stage**	pT1-pT2; pT3-pT4	5.76(3.49-9.48)	<0.001	1.036(0.42-2.56)	0.939
**Platinum resistance**	No ; Yes	7.702(4.31-13.8)	<0.001	6.41(2.67-15.37)	<0.001^§^
**Cytoreductive surgery**	Optimal ; suboptimal	3.08(1.78-5.33)	<0.001	0.85(0.41-2.09)	0.851
**Recurrent**	Yes; No	9.542(5.87-15.5)	<0.001	2.01(0.79-5.10)	0.141

Note: Cox proportional hazards regression was used to perform uni- and multivariant analysis for potentially important variants.

Abbreviations: HR, hazard ratio; CI, confidence interval.

§ Two-sided Cox proportional hazards regression using normal approximation.

### POSTN induces cisplatin resistance in A2780 cells through AKT activation

**Figure 4:**
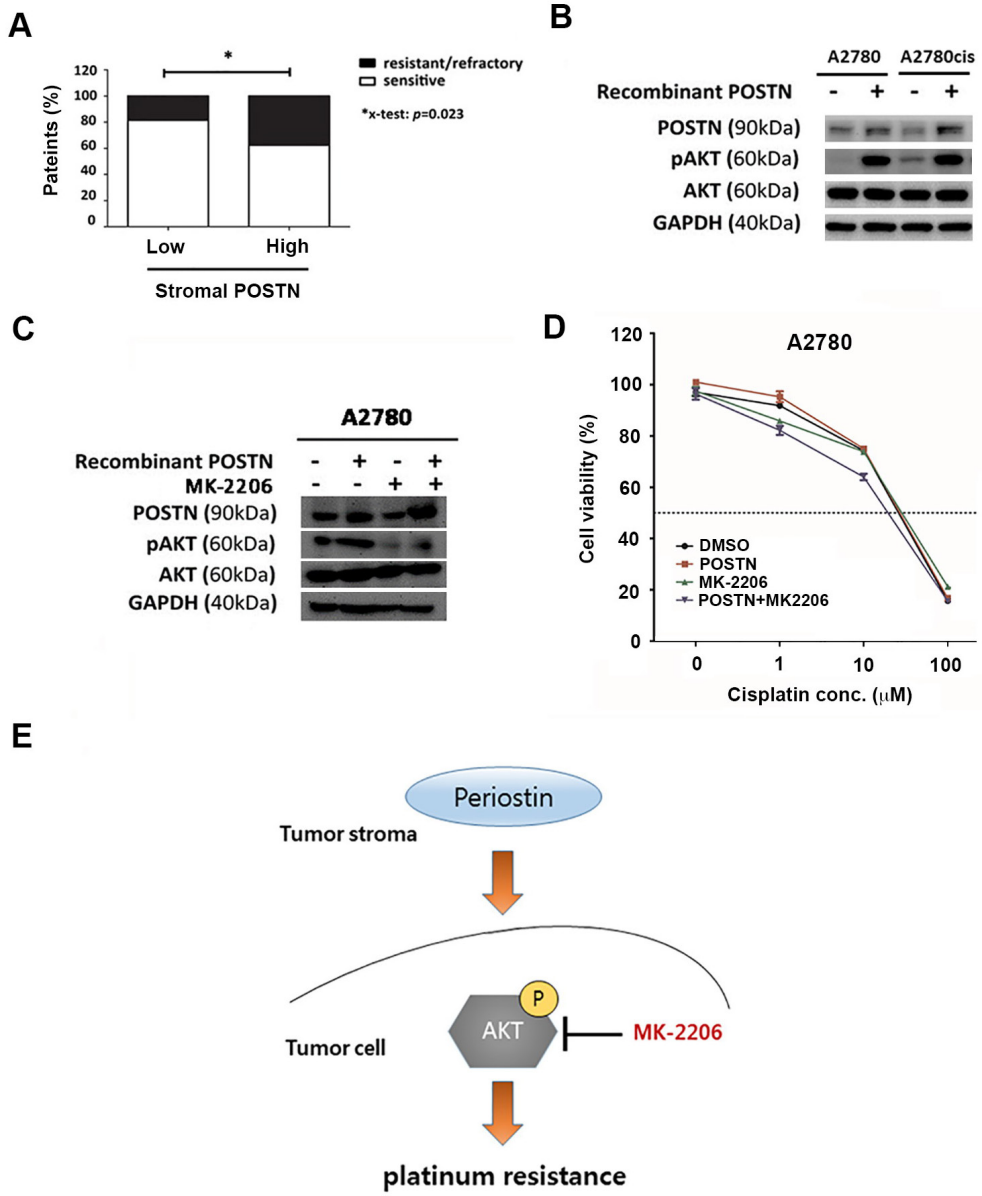
POSTN induces platinum resistance via PI3K/AKT pathway **A.** Bar chart represents percentage of platinum resistance in patients with high levels of stromal POSTN expression and those with low levels of stromal POSTN. **B.** Western blot analysis of POSTN, phosphor-AKT, total AKT, and GAPDH protein expression in A2780 cells and A2780 cisplatin-resistant cells. **C.** Western blot analysis of endogenous POSTN, phosphor-AKT, total AKT, and GAPDH protein expression in A2780 cells upon recombinant POSTN protein and/or MK-2206 treatment. **D.** Cytotoxicity assay of A2780 cells treated with cisplatin at indicated concentration in the presence or absence of recombinant POSTN (1μg/ml) or AKT inhibitor, MK-2206 (1 μM). Recombinant POSTN increased cisplatin IC_50_ of A2780 from 18 μM to 23 μM and then decreased to 14 μM after adding MK-2206. **E.** Schematic model shows the relationship between stromal POSTN and the development of platinum resistance in ovarian cancer. Periostin in cancer stroma can activate intracellular AKT-S473 phosphorylation to promote platinum resistance, which can be reversed by AKT inhibitor, MK-2206.

To determine the relationship between POSTN and platinum resistance, we analyzed a subset of 140 patients that received platinum therapy. Patients with resistance or refractory group were defined as those with disease recurrence within 6 months after primary platinum-based chemotherapy or those persist with diseases (*n* = 35; 25.0 %), whereas patients in platinum sensitive group were defined as those who were disease-free for more than 6 months after primary platinum-based chemotherapy (*n* = 105; 75 %) (Supplementary Table S1). Our results showed patients with high stromal POSTN expression had significantly higher percentage of platinum resistant patients compared to patients with low stromal POSTN (37.5 % versus 18.5 %, *p* = 0.023, Figure 4A). Cisplatin-resistant cell line A2780cis has increased pAKT then A2780 (Figure 4B). After adding POSTN, both cells line increased pAKT to similar intensity. To explore the effect of POSTN treatment on platinum sensitivity in A2780 epithelial ovarian cancer cells, we treated the A2780 cell line in 4 groups: (1) control group (2) POSTN group with recombinant POSTN protein (1 μg/ml) in cultured medium for 24 hours (3) AKT inhibitor group with MK-2206(1 μg/ml) for 2 hours (4) combined group with recombinant POSTN protein (1 μg/ml) in cultured medium for 24 hours followed bv AKT inhibitor groups with MK-2206(1 μg/ml) for 2 hours. Protein levels of endogenous POSTN in the A2780 cell line increased after treatment with recombinant POSTN, and was capable of phosphorylating Akt at serine 473 and inhibited by AKT inhibitor, MK-2206 (Figure 4C). Recombinant POSTN increased cisplatin IC_50_ of A2780 from 18 μM to 23 μM and then decreased to 14 μM after adding MK-2206 (Figure 4D). Taken together, the results revealed that stroma POSTN may induce resistance to cisplatin through activating Akt pathway and platinum resistance can be reversed by AKT inhibitor (Figure 4E).

## DISCUSSION

Nearly all of ovarian cancer patients received first-line platinum/taxane-based treatment regimen as the standard care. However, many patients are intrinsically resistant to platinum therapy. Therefore, biomarkers that can be used to predict platinum response are urgently needed. In this study, POSTN protein immunoreactivity observed in cancer stromal cells was found to be a predictive marker for platinum response and survival prognosis. Furthermore, the pathway involved in POSTN and platinum response in EOC may be related to the activation of AKT pathway. AKT inhibitor, MK-2206, can enhance the platinum response with patients with high POSTN expression in the tumor microenvironment, which may provide as a biomarker for clinical trial.

In the present study, the the mRNA expression levels in cancer stroma cells were the highest among those in tumor cells and normal ovarian tissues. The highest relative mRNA expressions of POSTN were observed in malignant adjacent cancer stromal area among distal regions and tumor cells. The clinical correlation of POSTN expression location in epithelial ovarian carcinoma cell or tumor stroma cells is still controversial. Zhu *et al*. showed that POSTN expression in tumor cells was significantly higher in advanced stage, high grade and recurrent tumors in 126 patients with primary and recurrent EOCs [14]. Recently, Choi *et al*. presented that POSTN was mainly expressed in cancer-associated stromal fibroblasts, but not in cancer cells and the expression level of POSTN in cancer stromal cells was highly correlated with FIGO staging, tumor mitosis, poor survival, distal metastasis, tumor recurrence of 66 ovarian cancer patient cohorts [20]. Similar findings were also reported in other types of cancer. In NSCLC, POSTN in cancer stroma cells is a prognostic factor for poor progression-free survival [27]. In prostate cancer, IHC analysis showed POSTN expression was up-regulated in high-grade prostate cancers and stromal POSTN was shown to be a progression factor for prostate specific antigen relapse-free survival [28]. Stromal expression of POSTN was also correlated with poor survival and bone metastasis in breast cancer [29]. Here, we showed that higher POSTN expression in cancer stromal cells, rather than in tumor cells, was significantly correlated with shorter disease-specific progression-free survival (PFS) and overall survival (OS) in multivariate analysis. Taken together, POSTN expression in cancer stromal cells can be served as a poor prognostic marker for epithelial ovarian cancer. However, in this study, tissue microarray contained different subtypes of epithelial ovarian cancer and borderline tumor and cannot distinguish which subtype of EOC is more correlated to POSTN expression due to limited numbers in different subtypes. EOC is a complex disease due to varieties of histological subtypes and different populations have variable distributions of those subtypes of EOC [30]. Further investigations are needed to determine the role of stromal POSTN in different subtypes of EOC.

In a previous study, tumor POSTN expression was shown to significantly promote intraperitoneal tumor metastatic growth in immunodeficient mice by increasing tumor angiogenesis and decreased tumor cell apoptosis [14]. Malanchi *et al*. reported that POSTN-deficient tumor cells of breast cancer cannot form tumor spheres and this can be rescued by adding POSTN protein in primary cultures. Stromal POSTN was found to connect the metastatic niche and cancer stem cell to create a cancer stem cell (CSC) -supportive niche and promote metastatic colonization by augmenting the Wnt signaling pathway [31]. Here, we found high levels of POSTN expression in cancer stroma was correlated with advanced disease stage, which is often associated with peritoneal and distal metastasis. Further study is needed to investigate the relationship between POSTN in tumor microenvironment and ovarian cancer stem cell properties.

Chien et al. showed carcinoma-associated mesenchymal stem cells (CA-MSC) secretions can promote AKT and XIAP phosphorylation and suggested that tumor microenvironment as well as the extracellular matrix (ECM) can alter tumor's response to chemotherapy [32]. POSTN was found to bind to the integrins αvβ3, αvβ5, and α6β4 to promote the recruitment of the epidermal growth factor receptor [33] or to active the Akt/PKB- and FAK mediated signaling pathways [8]. Ryner et al recently reported that POSTN, LOX (lysyl oxidase), and FAP(fibroblast activating protein) were the top 3 up-regulated genes within the peritumoral stromal, which were specifically associated with the clinical chemoresistance. They also reported high levels of POSTN could predict shorter progression-free survival following first-line chemotherapy by analysing on a set of 85 ovarian tumors as well as on an independent validation dataset containing 138 ovarian patients from the chemo-treatment arm of the ICON7 trial recurrent tumors [21]. Moreover, Ryner et al also revealed that ovarian tumor cells grown in the presence of recombinant POSTN protein promoted resistance to carboplatin and paclitaxel treatment *in vitro* [21]. Similar to our findings, we found POSTN treatment increased the resistance to cisplatin through AKT pathway and could be reversed by AKT inhibitor, MK-2006 (Figure 4C–4E). MK-2206 is an orally active allosteric Akt inhibitor that is under development for the treatment of solid tumors. MK-2206 is a highly potent and selective Akt inhibitor [34]. Some studies revealed that MK-2206 synergistically enhanced cell growth inhibition induced by chemotherapeutic agents in several ovarian cancer cell lines [34, 35]. In this study, we found that MK-2206 can decrease resistance to cisplatin caused by POSTN treatment and the results suggested that POSTN expression in cancer stroma may be an indicator for poor survival and platinum resistance and the use of MK-2206 may be beneficial to augment the efficacy of existing cancer therapeutics.

The alternate hypothesis for chemoresistance is that cancer cells can acquire extracellular matrix (ECM)-dependent platinum resistance [32]. Serial analysis of gene expression (SAGE) profiling of cisplatin-resistant and sensitive cells revealed many ECM genes expressions such as collage VI, are elevated in the resistant cells. Sensitive cells in the present of collagen VI protein promoted resistance. The other ECM protein, fibrillin-1, expressed in primary tumor are associated with early recurrence in platinum-sensitive ovarian cancer [36]. In the present study, POSTN can be stained either in stromal cell and cancer cells *in vivo* with positive correlation. By adding POSTN protein, we showed increased POSTN protein expression in tumor cells *in vitro*. Taken together, ECM component may play an important determinant for drug response.

In summary, we found that expression of POSTN in cancer stromal cells, rather than in tumor cells, not only serves as a prognostic factor for clinical outcome but also an important indicator for platinum response in EOC patients. The resistance to cisplatin induced by tumor environment POSTN may be dependent on tumor intrinsic PI3K/AKT pathway, which provides a window of opportunity for reversing platinum resistance by targeting AKT.

## MATERIALS AND METHODS

### Gene expression analysis of tumor and tumor environment in EOC

To evaluate mRNA level of POSTN in normal stroma cells, cancer stroma cells, normal ovarian surface epithelial cells, and cancer epithelial cells, we analyzed the microarray data retrieved from GSE38666 (http://www.ncbi.nlm.nih.gov/geo/query/acc.cgi?acc=GSE38666) and GSE29156 (downloaded from http://www.ncbi.nlm.nih.gov/geo/query/acc.cgi?acc=GSE29156) of the NCBI GEO database. The GSE38666 microarray data contains gene expression datasets of 8 normal stroma (NS) and 8 matched normal ovarian surface epithelium (OSE) from 12 individuals, along with 7 cancer stroma and 7 matched cancer epithelium from 18 additional ovarian cancer patients. The GSE29156 microarray data contains 23 tissue samples laser capture micro-dissected from an ovary with a benign serous tumor (specifically 4 normal epithelium samples, 5 tumor samples, 6 stroma samples adjacent to the tumor, and 8 stroma samples distal of the tumor), and 27 tissue samples laser capture micro-dissected from an ovary with a malignant serous tumor (specifically 5 normal epithelium samples, 8 tumor samples, 7 stroma samples adjacent to the tumor, and 7 stroma samples distal of the tumor).

### Patients and tumor samples

Tumor tissues in paraffin blocks of 308 female patients diagnosed with epithelial ovarian adenocarcinoma and borderline ovarian cancer were obtained from the surgical archives of the Department of Pathology and Department of Gynecology and Obstetrics, Taipei Veteran General hospital, and the surgical archives of the Department of Pathology and Department of Gynecology and Obstetrics, Kaohsiung Chang Gung Memorial Hospital. The time of diagnosis of the patients from the Taipei Veteran General hospital was between 2000 to 2008, and that of Kaohsiung Chang Gung Memorial Hospital was between 2000 to 2007.

Clinical stage after debulking surgery was determined using the criteria established by the International Federation of Gynecology and Obstetrics (FIGO staging system for ovarian cancer 2009) [37]. Clinical data including age, cytoreduction status (optimal vs. suboptimal), type of chemotherapies, overall survival (OS) and disease-free survival (DFS) were retrieved from medical records. The duration of OS was measured from the date of diagnosis to death or censored at the date of last follow-up. The DFS was measured as the duration between the dates of diagnosis to disease recurrence. Cytoreductive operations include total abdominal hysterectomy, bilateral salpingo-oophorectomy, infra-omentectomy, appendectomy, and pelvic and para-aortic lymph node sampling and were performed in all patients except in 9 of 14 patients with borderline ovarian tumor who received only unilateral salpingo-oophorectomy [38]. Optimal surgical cytoreduction was defined as operation for residual tumor ≤1 cm in diameter and suboptimal surgery was defined as operation for residual tumor > 1 cm in diameter [39]. Standard adjuvant chemotherapy including platinum and paclitaxel regimen was given to 283 patients after surgery except in 9 patients with borderline tumor and 16 patients with low risk early stage disease, who could not tolerate the chemotherapy or had persist tumor after suboptimal cytoreductive surgery” change to “16 patients with low risk early stage disease or who could not tolerate the chemotherapy. A clinical remission was defined as no palpable tumor and normal CA-125 levels after the completion of chemotherapy. Patients were then followed up every 3 months during the first 5 years, and then every half year thereafter. Recurrence was defined as elevation of CA-125 or pathologically proved adenocarcinoma after complete recovery from the cancer. Surgery or chemotherapy was initiated when relapse occurred. Sensitivity to primary platinum-based chemotherapy was defined as recurrence after 6 months after completing primary platinum-based regimen, and resistant or refractory was defined as recurrence within 6 months after primary platinum therapy or persist/stable diseases [3].

The ovarian cancer tissue samples obtained at the primary surgery were stained with hematoxylin and eosin and reviewed to confirm the histopathologic diagnosis and tumor grading. All specimens were collected under protocols approved by the institutional review board of both hospitals.

### Tissue array construction

The tissue arrays were constructed with a total of 308 primary epithelial ovarian cancers, which included 99 serous adenocarcinoma, 61 endometrioid adenocarcinoma, 63 clear cell adenocarcinomas and 48 mucinous carcinomas. Fourteen borderline tumors were also included. Twenty-three transitional cell carcinomas or mixed type were grouped into the histological subtype as others. Paired normal tissues were arrayed adjacent to the corresponding cancerous tissues as described previously [40]. Matched H&E reference slides were cut from all donor blocks and graded, and targeted tissue types of interest were marked. For the tissue array construction, core tissue biopsies (diameter 1 mm; height 3-4 mm) were taken from paraffin-embedded tumor blocks and were precisely arrayed into a new recipient paraffin block (27 × 22 mm). Tissue core biopsies were subsequently punched from selected regions of the donor blocks using a thin-wall, stainless steel tube that was sharpened similar to a corkscrew. Once the TMA block had been prepared, the blocks were sectioned into 4-μm thick sections, stained with H&E for use as a reference to confirm the histology, and subsequently treated with target immunohistochemical stains.

### Immunohistochemistry

The formalin-fixed, paraffin-embedded tissue sections (5 μm) were deparaffinized in xylene and rehydrated in graded concentrations of ethanol. Antigen retrieval was enhanced by heating the slides at 121°C for 5 minutes in DAKO target retrieval buffer (pH 6.1) using a pressurized decloaking chamber (Biocare Medical). Endogenous peroxidase was blocked with 3% hydrogen peroxide followed by normal goat serum (10%) blocking for 1 hour. Sections were next incubated at 4°C for 12 hours with primary anti-human POSTN (rabbit polyclonal, 1:250 dilution, Genetex, Taiwan). Sections were rinsed with PBS twice and incubated for 1 hour with biotinylated secondary antibodies followed by 30-min incubation at room temperature with horseradish-peroxidase-conjugated streptavidin. Immunostaining was developed with DAB (3-3′ diaminobenzidine tetrahydrochloride) and counterstained with light hematoxylin. The sections were examined by light microscopy. A four-point staining intensity scoring system was used for determining the relative expression of POSTN in cancer specimens and related stromal; the staining expression was semiquantatively and arbitrarily score ranged from 0 (negative expression), 1 (weak expression), 2 (moderate expression) to 3 (strong expression) modified from Choi et al [20] (designed as 0,no staining or less than 10% of stromal or tumor cells ; 1, weak, 10-30% of stromal or tumor cells ; 2, 30-60% of stromal or tumor cells; 3, more than 60% of stromal or tumor cells). All of the immunohistochemical staining results were reviewed and scored by two independent pathologists."

### Cell line, chemicals and reagents

Human ovarian adenocarcinoma A2780/A2780cis cells were purchased from The European Collection of Cell Cultures (ECACC). All cell culture reagents were purchased from Invitrogen (Taiwan). Recombinant human POSTN protein and anti-POSTN antibody were purchased from R&D Systems (Minneapolis, MN). Anti-glyceraldehyde-3-phosphate dehydrogenase (GAPDH) antibody and primary antibodies against AKT, phospho-AKT (p-AKTSer473) were purchased from Cell Signaling Technology (Danvers, MA, USA). Cisplatin (*cis*-Diammineplatinum(II) dichloride) and AKT inhibitor MK-2206 were purchased from Adooq Bioscience (Irvine, CA, USA).

### Cell proliferation assay

Human epithelial ovarian cancer A2780 cells were seeded in 96-well plates at a density of 10000 cells/well in culture medium. Following overnight incubation, control groups did not receive periostin nor AKT inhibitor. Recombinant periostin protein (1μg/ml) was added for 24 hours in the periostin group. MK-2206 (1μM) cultured for 2 hours was MK-2206 group. Recombinant periostin protein (1μg/ml) was added for 24 hours followed by MK-2206 (1μM) for another 2 hours was added in the combined group. Then 3 sets of cells were exposed to a range of drug concentrations of cisplatin. Cell proliferation was measured with the SRB assay [41] and IC50 were analyzed according to the method of Chou and Talalay [42] by using the CompuSyn software program downloaded from http://www.combosyn.com/.

### Western blotting analysis

Cells treated by indicated conditions were pelleted by centrifugation and rinsed with PBS. The cell pellets were then lysed in RIPA buffer. DNA in the lysate was sheared by a sonicator. Concentration of protein was determined using Lowry assays (Bio-Rad). Protein extracts were loaded onto SDS-PAGE and were transferred to nitrocellulose membranes (Bio-Rad Laboratories, Inc.). The membranes were analyzed by specific primary (p-AKT, AKT, POSTN and GAPDH) and secondary antibodies. The signals were developed using an ECL chemiluminescence kit (Amersham Biosciences, UK).

### Statistical analysis

Statistical analysis was carried out using the PASW computer program package (PASW Statistics v18, Chicago, IL, USA). The association between POSTN expression cancer stroma and tumor cell expression and clinicopathological parameters and drug response was evaluated using the Fisher's exact test. Univariate survival analysis was based on the Kaplan–Meier method. Comparison between the survival curves was analyzed using the Log-rank or the Breslow test. The prognostic significance of POSTN expression in cancer stroma or cancer cell expression concerning other pathological variables was assessed using the multivariate Cox's proportional hazard's analysis. A value of *p* < 0.05 was considered statistically significant.

## SUPPLEMENTARY TABLE


