# Supplementary material for: Periostin in tumor microenvironment is associated with poor prognosis and platinum resistance in epithelial ovarian carcinoma
Source: Oncotarget. 2015 Dec 21;7(4):4036–47. doi: 10.18632/oncotarget.6700 (PMC4826188; doi:10.18632/oncotarget.6700)
Supplement: Supplementary file 1 [file oncotarget-07-4036-s001.pdf]

Supplementary Table S1: POSTN expression in cancer stroma cell and platinum treatment response

Chemotherapy response	Sensitivity to platinum$^{\Phi}$ (n = 105; 75.0%)	Resistance to platinum§ (n = 35; 25.0%)	*P*-value
High expression of stromal cell	30 (62.5)	18(37.5)	0.023*
Low expression of stromal cell	75(81.5)	17(18.5)	

$^{\Phi}$Sensitivity to platinum defined as tumor recurrence after 6 months after completing primary platinum therapy
§Resistance to platinum defined as tumor recurrence before 6 months after completing primary platinum therapy or refractory to platinum resistance
*Two-sided Fisher's exact test
